# Paromomycin Affects Translation and Vesicle-Mediated Trafficking as Revealed by Proteomics of Paromomycin –Susceptible –Resistant *Leishmania donovani*

**DOI:** 10.1371/journal.pone.0026660

**Published:** 2011-10-27

**Authors:** Bhavna Chawla, Anupam Jhingran, Aswini Panigrahi, Kenneth D. Stuart, Rentala Madhubala

**Affiliations:** 1 School of Life Sciences, Jawaharlal Nehru University, New Delhi, India; 2 Seattle Biomed, Seattle, Washington, United States of America; Weill Cornell Medical College, United States of America

## Abstract

*Leishmania donovani* is a protozoan parasite that causes visceral leishmaniasis (VL) and is responsible for significant mortality and morbidity. Increasing resistance towards antimonial drugs poses a great challenge in chemotherapy of VL. Paromomycin is an aminoglycosidic antibiotic and is one of the drugs currently being used in the chemotherapy of cutaneous and visceral leishmaniasis. To understand the mode of action of this antibiotic at the molecular level, we have investigated the global proteome differences between the wild type AG83 strain and a paromomycin resistant (PRr) strain of *L. donovani*. Stable isotope labeling of amino acids in cell culture (SILAC) followed by quantitative mass spectrometry of the wild type AG83 strain and the paromomycin resistant (PRr) strain identified a total of 226 proteins at ≥95% confidence. Data analysis revealed upregulation of 29 proteins and down-regulation of 21 proteins in the PRr strain. Comparative proteomic analysis of the wild type and the paromomycin resistant strains showed upregulation of the ribosomal proteins in the resistant strain indicating role in translation. Elevated levels of glycolytic enzymes and stress proteins were also observed in the PRr strain. Most importantly, we observed upregulation of proteins that may have a role in intracellular survival and vesicular trafficking in the PRr strain. Furthermore, ultra-structural analysis by electron microscopy demonstrated increased number of vesicular vacuoles in PRr strain when compared to the wild-type strain. Drug affinity pull-down assay followed by mass spectrometery identified proteins in *L. donovani* wild type strain that were specifically and covalently bound to paromomycin. These results provide the first comprehensive insight into the mode of action and underlying mechanism of resistance to paromomycin in *Leishmania donovani*.

## Introduction

Paromomycin is an aminoglycosidic antibiotic which was initially used against bacterial infection [1]. It is also used against giardiasis, amoebaiasis [2] and cryptosporidiosis [3]. The mechanism of action of paromomycin has been well studied in *E. coli*. It is known to inhibit protein synthesis by interacting with the ribosomal subunits [1]. It has also been shown to inhibit the anti-association activity of initiation factor 3 and promote association of the ribosomal subunits [4]. It is known to bind to the major groove in the A-site of 16S rRNA in *E. coli* and induces misreading of mRNA [5].

The protozoan parasite *Leishmania* is the causative agent of kala-azar and is responsible for a variety of clinical manifestations. Visceral leishmaniasis (VL) is caused by *L. donovani* in the Indian sub-continent. Pentavalent antimonials (SbV) are the first line of drug used in the treatment against all forms of leishmanial infections [6], [7]. Resistance to this drug has become a major barrier in the treatment of VL in many endemic regions particularly in India [8]. A parenteral formulation of aminosidine (paromomycin) has been approved for leishmaniasis treatment in India [9]–[11], where it is in phase IV trials (http://www.oneworldhealth.org/press_releases/release/pr_1227120528). It has proved to be useful against cutaneous (as both topical and parenteral formulation) and visceral leishmaniasis (as parenteral formulation) [12], [13].

The mode of action of paromomycin is not clear in case of *Leishmania*. It has been proposed that it might alter membrane fluidity, interact with ribosomes, interfere with the mitochondrial membrane potential and inhibit respiration [14]. We had earlier elucidated the effect and mechanism of uptake of paromomycin in *Leishmania donovani* [15]. A line selected for resistance to the drug showed reduced paromomycin accumulation associated with a significant reduction in the initial binding to the cell surface. The drug induced reduction in membrane potential and inhibition of protein synthesis were less pronounced in the resistant strain in comparison to the wild-type [15]. Recent report indicates differential effects of paromomycin on the translation processes of the *Leishmania* parasite and its mammalian hosts [16].

Drug resistance is a multifactorial problem due to changes in the expression levels and activity of a wide number of proteins. Quantification of mRNA levels between drug resistant and drug sensitive cell lines unfortunately do not always correlate with protein expression levels due to post-transcriptional changes in protein abundance. Therefore global quantitative proteomics screens are needed to identify the protein targets that are differentially expressed in drug resistant cell lines. Protein profiling has previously been applied to understand the stage- specific gene expression, drug resistance mechanism, identification of virulence factors and characterization of immunodominant antigens [17]–[20]. Earlier reports on comparative protein profiling of the wild type and the antimonial-resistant strain showed that the heat shock protein and kinetoplastid calpain related proteins modulate susceptibility to antimonials [21]. In another study novel roles were revealed for methionine adenosyl transferase in methotrexate resistance in *Leishmania* [18].

In order to understand the mode of action and possible mechanism of resistance of this antibiotic at the molecular level, we have investigated the protein expression profile of genetically related pair of paromomycin susceptible/-resistant strains. A quantitative proteomic approach based on stable isotope labeling of amino acids in cell culture (SILAC) followed by high resolution mass spectrometry was employed to analyze the differences in the proteome of the wild type and the PRr resistant strain. Paromomycin- resistant promastigotes were generated previously under step-wise exposure to paromomycin and were found to display a three-fold increase in resistance compared to the wild-type [15]. Drug affinity pull-down assay followed by mass spectrometery revealed a number of proteins in *L. donovani* which might be interacting with paromomycin. Internalization probably then appears to proceed by endocytosis as reported in our earlier studies [15]. Upregulation of proteins involved in vesicular trafficking in the PRr strain further supports sequestration of drug in the vesicular cytoplasmic compartment. Ultrastructural studies demonstrated increased number of vesicular vacuoles in the PRr strain when compared to the wild-type strain. Up-regulation of proteins involved in the translational machinery especially the ribosomal proteins in the PRr strain indicates that once into the cell PR inhibits protein synthesis by targeting the ribosomal proteins. The identified parasite proteins provide an insight in to the mode of action and underlying mechanism of resistance to paromomycin in *Leishmania.* Furthermore, it allowed us to reinterpret and extend earlier findings, identifying additional processes hitherto only suspected to be involved in its mode of action and underlying mechanism of resistance in *L. donovani.*

## Results

### Identification of Proteins Differentially Expressed in Wild Type (WT) and the Paromomycin Resistant (PRr) Strain

**Figure 1:**
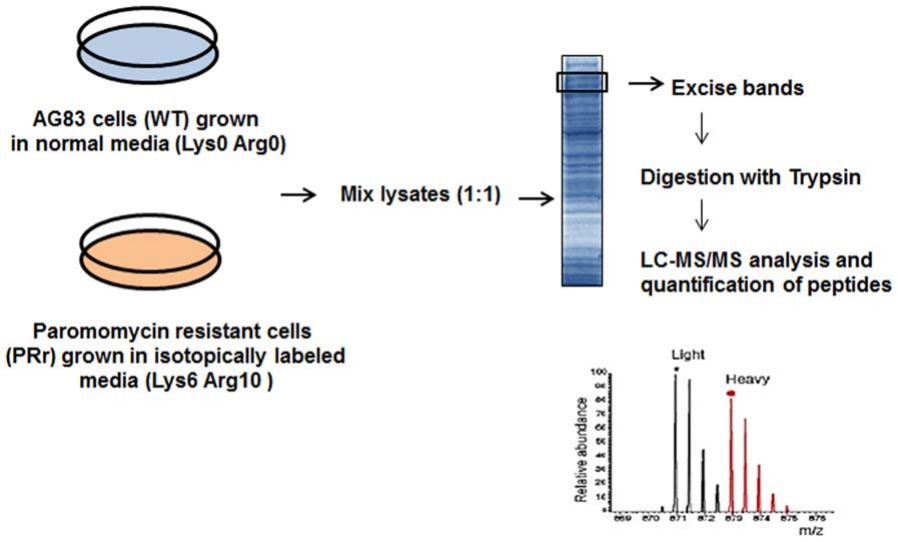
Experimental outline of the SILAC experiment. Paromomycin resistant cells (PRr) and AG83 wild type (WT) were grown on isotopically heavy and light media respectively. The cells from both the strains were lysed and light and heavy cell lysates were combined. Protein samples were reduced, alkylated and digested with trypsin into peptide samples that were then analyzed by LC-MS/MS. Quantification of SILAC peptide pairs was performed with the SEQUEST algorithm against *L. infantum* protein database ver 3.0 (ftp://ftp.sanger.ac.uk/pub/pathogens/L_infantum/). The SEQUEST output files were analyzed and validated by PeptideProphet.

**Figure 2:**
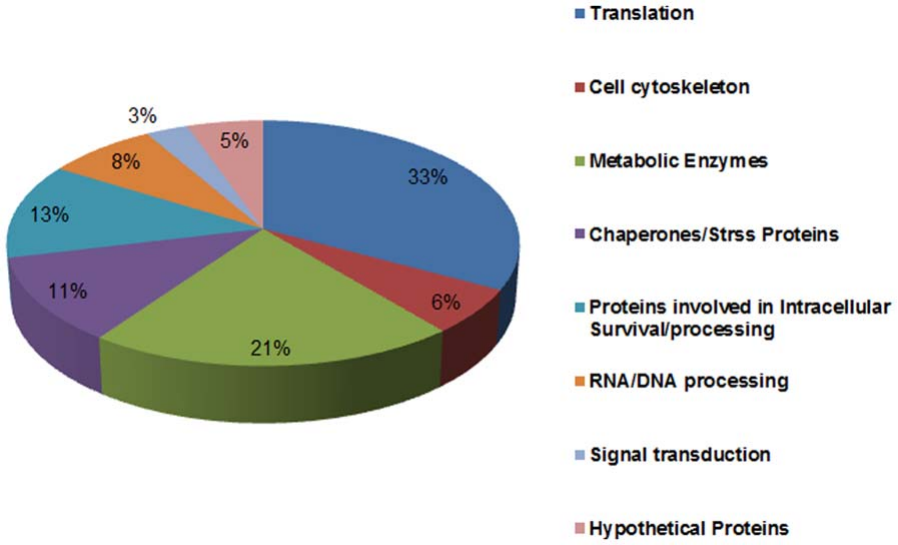
Pie diagram showing distribution of *Leishmania* proteins identified by quantitative proteomics technique using stable isotope labeling with amino acids in cell culture (SILAC) coupled with mass spectrometry to quantify changes in protein levels between paromomycin resistant (PRr) and sensitive cells. Proteins were grouped according to their cellular functions. The up- and down- regulated hits are combined in each group.

We have compared the proteome of the wild type (WT) strain, AG83-S with the PRr strain. Comparative proteomic studies using SILAC technology was employed to reveal proteins that are involved in mediating drug resistance. Briefly, the WT and the PRr strains were cultured in the medium supplemented with normal amino acids [L-lysine (70 mg/L) and L-arginine (70 mg/L)] and isotopically labeled ^13^C_6_ L-lysine-HCl and ^13^C_6_^15^N_4_ L-arginine-HCl respectively. A schematic diagram illustrating the experimental outline is shown in Figure 1. Equal amount of protein (100 µg) from the sensitive and the resistant strains were mixed and subjected to the first dimension chromatography and the peptide masses obtained from mass-spectrometry analysis were characterized using the *L. infantum* V3 proteome version 3.0 (ftp://ftp.sanger.ac.uk/pub/pathogens/L_infantum/). Supplementary Table S1 in the supporting information includes all the protein IDs with ≥95% confidence from two individual datasets with their respective ratios and p-values. A total of 226 distinct proteins were identified out of which 197 proteins had a known or predicted function and 29 proteins had no known function (hypothetical proteins). Supplementary Table S2 includes merged datasets and functional annotation of all the non-redundant proteins identified at ≥95% confidence and ≥2 peptides. According to the functional description, all significantly identified proteins fell into 7 major groups: cell cytoskeleton, metabolic enzymes, chaperones/stress proteins, translation, proteins involved in intracellular survival, RNA and DNA processing, signal transduction and hypothetical proteins. The relative distribution of proteins is shown in Figure 2. The up- and down- regulated hits are combined in each group.

**Figure 3:**
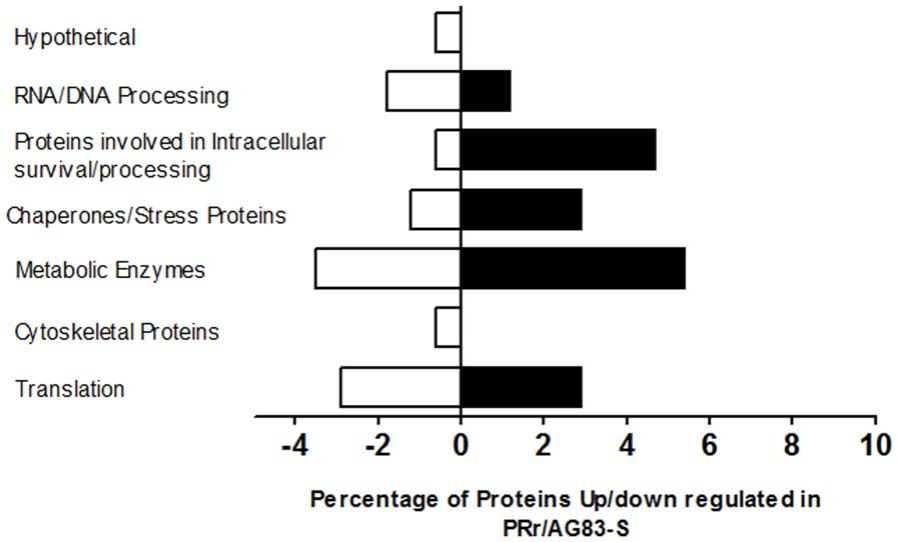
Bar diagram showing the proteins that were up/downregulated in PR resistant strain. The proteins have been grouped according to cellular functions based upon distinct peptides quantitated at ≥95% confidence and having ≥2 peptides.

Among the strictly quantitated proteins, we defined the following thresholds for selecting proteins that had significantly altered expression: 1) proteins identified by the software having ≥95% confidence; 2) the ratio is either ≥1.5 or ≤0.5 and 3) proteins having ≥2 peptides. Applying these filtering criteria, subsets of regulated proteins were identified and then the total number of protein IDs and the number of regulated hits (meeting all three requirements) found in the experiment was determined as detailed in the methods section. The proteins which were ≥1.5 fold were considered to be upregulated whereas the proteins which were ≤0.5 fold were considered to be down regulated. Using these stringent parameters, 29 proteins were found to be up-regulated and 21 proteins were found to be down-regulated and rest of the proteins remained unchanged in the PRr strain in comparison to the wild type (Table 1 and Table 2). Figure 3 shows the percentage of up- and down regulated proteins based on their functional classification.

**Table 1:** Proteins upregulated in the paromomycin resistant (PRr) strain.

S. No.	Accession No.	Name of the Protein(Up-regulated)	PRr:WT ratio	P-value	Percent Coverage	Total Number of Peptides
**Translation**						
1	LinJ15_V3.1530	Ribosomal protein S6, putative	1.57±0.4	4.21E-01	34.9	6
2	LinJ28_V3.0570LinJ30_V3.3240	Ribosomal protein s26, putative	3.47±0.6	2.05E-02	15.2	2
3	LinJ28_V3.1050LinJ30_V3.3650	40S ribosomal protein S14	1.65±1	5.39E-01	29.2	14
4	LinJ32_V3.0930LinJ35_V3.0600	60S ribosomal protein L18a, putative	1.56±0.3	4.07E-01	6.7	3
5	LinJ36_V3.0210	Elongation factor 2	1.5		58.8	5
**Metabolic Enzymes**						
**Glycolysis**						
6	LinJ14_V3.1240	Enolase	2.4		67.8	9
7	LinJ21_V3.0300LinJ21_V3.0310	Hexokinase, putative	1.5±1.1	6.52E-01	10.6	8
8	LinJ24_V3.0870	Triosephosphate isomerase	1.51±0.5	5.06E-01	10.8	9
9	LinJ30_V3.2990LinJ30_V3.3000	Glyceraldehyde 3-phosphate dehydrogenase, glycosomal	1.94±0.6	2.68E-01	30.2	138
10	LinJ36_V3.6960	2,3-bisphosphoglycerate-independent phosphoglycerate mutase	1.57±0.6	4.85E-01	5.6	2
**Other Metabolic Enzymes**						
11	LinJ16_V30560	Orotidine-5-phosphate decarboxylase/orotate phosphoribosyltransferase, putative	1.56±0.5	4.66E-01	12	21
12	LinJ24_V3.0780	Malic enzyme	1.78±1.5	5.52E-01	4.6	3
13	LinJ27_V3.0300	Acyl carrier protein, putative	1.78±0.2	2.67E-01	12	4
14	LinJ30_V3.3560LinJ30_V3.3580	S-adenosylmethionine synthetase	1.63±0.07	3.38E-01	2.3	2
**Chaperones/Stress Proteins**						
15	LinJ26_V3.1220	Heat shock protein 70-related protein	2.11±0.2	1.50E-01	15.8	7
16	LinJ28_V3.3060	Heat-shock protein hsp70, putative	2.5467		62.5	10
17	LinJ30_V3.2480	Heat shock 70-related protein 1, mitochondrial precursor, putative	2.01		49.7	3
18	LinJ32_V3.3470	Chaperonin alpha subunit, putative	5.75±0.4	6.65E-04	7.9	5
19	LinJ36_V3.7240	Chaperonin, putative	2.26±0.2	1.16E-01	7.8	6
**DNA/RNA Processing**						
20	LinJ35_V3.2240	RNA-binding protein, putative	1.5±0.7	5.71E-01	12.4	8
21	LinJ36_V3.3960	Basic transcription factor 3a, putative	2.68±0.9	1.07E-01	35	17
**Proteins Involved in Intracellular Survival/Processing**						
22	LinJ11_V3.0350	14-3-3 protein, putative	1.64±0.6	4.41E-01	13.4	7
23	LinJ12_V3.0730	NADH:flavin oxidoreductase/NADH oxidase, putative	2.89±2.7	3.13E-01	9	5
24	LinJ19_V3.0150	Aminopeptidase, putative	1.73±0.2	2.93E-01	11.1	6
25	LinJ20_V3.0820	Vesicle-fusing ATPase, putative	2.8±0.4	5.21E-02	6.1	3
26	LinJ24_V3.1560LinJ24_V3.1570	IgE-dependent histamine-releasing factor, putative	1.75±0.2	2.88E-01	5.3	2
27	LinJ28_V3.2610	Vacuolar ATP synthase subunit B, putative	1.64±1.2	5.75E-01	4.8	2
28	LinJ29_V3.0790	Lipophosphoglycan biosynthetic protein, putative	2.01±0.2	1.77E-01	5.6	3
29	LinJ36_V3.3360	14-3-3 protein-like protein	1.56±0.5	4.59E-01	22.9	10

List of proteins with increased expression levels in the PR resistant strain.

Fold change of protein level measured by SILAC with standard deviation is given as PRr:WT ratio.

p-value determined here were analyzed and validated by PeptideProphet.

**Table 2:** Proteins downregulated in the paromomycin resistant (PRr) strain.

S. No.	Accession No.	Name of the Protein(Down-regulated)	PRr:WT ratio	P-value	Percent coverage	Total Number of Peptides
**Translation**						
1	LinJ11_V3.1160	Eukaryotic release factor 3, putative	0.17	1.45E-03	5.3	3
2	LinJ18_V3.0630LinJ36_V3.3950	60S ribosomal protein L10a, putative	0.24±0.2	1.99E-01	22	5
3	LinJ22_V3.1370LinJ22_V3.1410	40S ribosomal protein L14, putative	0.23±0.1	8.20E-03	5.2	4
4	LinJ30_V3.0470	Aspartyl-tRNA synthetase, putative	0.09±0.01	8.88E-06	3.6	3
5	LinJ35_V3.2040	60S ribosomal protein L32	0.27±0.2	1.88E-01	31.6	5
Cell cytoskeleton						
6	LinJ14_V3.1190	Kinesin K39, putative	0.3±0.04	2.40E-02	7.3	2
**Metabolic Enzymes**						
**Glycolysis**						
7	LinJ20_V3.0110LinJ20_V3.0120	Phosphoglycerate kinase C, glycosomal	0.1±0.1	6.67E-02	19.1	14
**Krebs cycle**						
8	LinJ35_V3.0850	NADH-dependent fumarate reductase-like protein	0.47±0.07	1.62E-01	2.3	3
9	LinJ35_V3.1190	NADH-dependent fumarate reductase, putative	0.24±0.02	5.57E-03	8.5	6
**Electron Transport Chain**						
10	LinJ05_V3.0500LinJ05_V3.0510	ATPase alpha subunit	0.45±0.27	3.12E-01	18.8	16
11	LinJ12_V3.0620	Cytochrome c oxidase subunit iv	0.25±0.28	2.61E-01	7.4	2
**Other Metabolic Enzymes**						
12	LinJ31_V3.2320	3,2-trans-enoyl-CoA isomerase, mitochondrial precursor, putative	0.39±0.03	6.69E-02	8.8	2
13	LinJ35_V3.3390	6-phosphogluconate dehydrogenase, decarboxylating putative	0.36±0.03	4.79E-02	8.6	3
**Chaperones/Stress Proteins**						
14	LinJ21_V3.1330	t-complex protein 1, delta subunit, putative	0.47±0.03	1.40E-01	12	4
15	LinJ35_V3.3900	t-complex protein 1, eta subunit, putative	0.42±0.1	1.09E-01	15.6	6
**DNA/RNA Processing**						
16	LinJ15_V3.1500	Proliferative cell nuclear antigen (PCNA), putative	0.27±0.1	2.87E-02	11.6	3
17	LinJ19_V3.0090	Fibrillarin, putative	0.45±0.1	1.69E-01	12.2	2
18	LinJ32_V3.0410	ATP-dependent RNA helicase, putative	0.27±0.2	1.58E-01	11.9	6
**Proteins Involved in Intracellular Survival/Processing**						
19	LinJ35_V3.0070	Prohibitin, putative	0.4±0.03	7.79E-02	7.9	2
**Hypothetical Proteins**						
20	LinJ10_V3.0910	Hypothetical protein, conserved	0.47±0.1	1.48E-01	12.8	2
21	LinJ35_V3.0140	Hypothetical protein, conserved	0.22±0.2	1.61E-01	21.8	3

List of proteins with decreased expression levels in the PR resistant strain.

Fold change of protein level measured by SILAC with standard deviation is given as PRr:WT ratio.

p-value reported here were analyzed and validated by PeptideProphet.

### Increased Glycolysis and Energy producing Metabolic Pathways in the Paromomycin Resistant strain (PRr)

*Leishmania* mainly uses glycolytic and pentose phosphate pathway enzymes for energy generation. Several enriched biological pathways from the subset of proteins identified with increased expression in the paromomycin resistant *L. donovani* are related to glycolysis. The enzymes of the glycolytic pathway were found to be significantly upregulated in the PRr strain except for the enzyme phoshoglycerate kinase which was found to be downregulated (∼10 fold) in the PR resistant strain. Other proteins, such as NADH dependent fumarate reductase putative and NADH dependent fumarate reductase like protein were also found to be downregulated (∼4 and ∼2 fold respectively) in the paromomycin resistant cells. This enzyme is involved in the conversion of fumarate to succinate and enables the organism to switch to anaerobic respiration. The protein 6-phosphogluconate dehydrogenase, the first committed enzyme of the pentose phosphate pathway was also found to be down regulated (∼2.7 fold) in the paromomycin resistant parasites. This indicates that the PRr strain heavily relies on glycolysis for its energy requirements. Increased glycolytic activity under normal aerobic conditions is probably associated with mitochondrial dysfunction. A high glycolysis rate also helps reduce oxidative stress because the product of anaerobic glycolysis, pyruvate, is a scavenger of hyperoxide.

Additional enriched metabolic pathways with enzymes displaying altered expression levels include the TCA cycle and lipid metabolism. Proteins involved in oxidative phosphorylation, ATPase alpha subunit and cytochrome c oxidase subunit IV were found to be downregulated in the PRr strain. Acyl carrier protein, which is a part of Type II fatty acid synthesis pathway, was found to be upregulated (∼1.8-fold) in the PRr strain. This pathway is used for lipoic acid biosynthesis which is an essential co-factor for the mitochondrial enzymes pyruvate dehydrogenases and 2-keto glutarate dehydrogenase present in the TCA cycle [22]. Another enzyme, S-adenosyl methionine synthetase was found to be ∼1.6 upregulated in the PRr strain. S-adenosyl methionine dependent RNA methylase, which methylates 16S rRNA in *E. coli* has been associated with resistance to kasugamycin, an inhibitor of protein synthesis [23].

### Modulation of Proteins Involved in Intracellular Survival, Processing and Virulence

The vesicular sequestration of drugs is a well known mechanism of drug resistance in some mammalian cell lines which results in lower cytoplasmic drug levels by sequestration of drugs in to a vesicular cytoplasmic compartment [24]–[26]. Drug resistance by sequestration in the vesicles has also been proposed for the antimony resistant *Leishmania* parasites. In *Leishmania*, trivalent antimony conjugates to trypanothione and is sequestered inside a vacuole by ABC (ATP binding cassette) transporter, P glycoprotein A (PGPA) [27]. We observed upregulation of both vesicle-fusing ATPase (∼2.8 fold) and vacuolar ATP synthase subunit B (∼1.6 fold) in the PRr strain.

**Figure 4:**
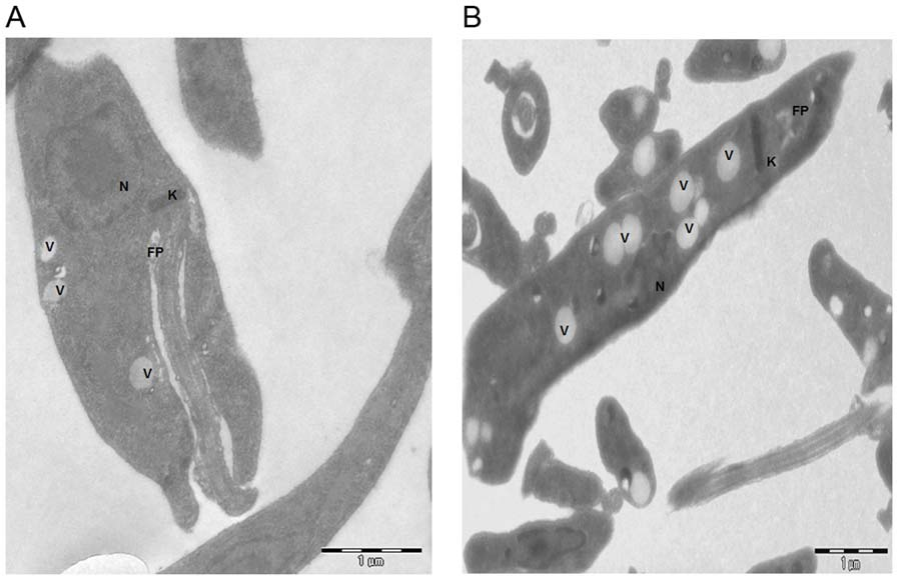
Transmission Electron Microscopic (TEM) images of the A. Wild Type (WT) promastigotes and B. Paromomycin resistant promastigotes. WT promastigotes were harvested and fixed as mentioned in Materials and methods and subjected to TEM analysis. PRr strain displayed numerous vacuoles compared to the wild type strain. Images are representative of 2 independent experiments. FP: Flagellar Pocket; V: Vacuole; N: Nucleus; K: Kinetoplast.

Transmission electron microscopy was employed in order to document the differences between the wild type and the PRr strain. The ultra-structural analysis of the paromomycin resistant strain revealed a greater number of vesicular vacuoles when compared to the sensitive wild type strain (Figure 4A and B). It is possible that vesicular sequestration of paromomycin into vacuoles by these resistant parasites might be involved in conferring the resistance phenotype.

We also observed increased expression of proteins that are involved in intracellular survival and virulence including lipophosphoglycan biosynthetic protein (LPG) putative, aminopeptidase IgE-dependent histamine releasing factor and NADH oxidase putative. Another protein, prohibitin, showed decreased protein expression levels (∼2.5 fold) in the PRr resistant strain. Prohibitin has been reported to play an important role in the events leading to *Leishmania-*host interaction [28]. Its homolog has been studied in *Trypanosoma brucei* and has been reported to be upregulated upon induction of apoptosis [29]. Down regulation of prohibitin in the present study possibly indicates that the PR resistant cells are able to survive by suppressing the expression of pro-apoptotic proteins. Two proteins, annotated as “14-3-3 protein like protein” and “14-3-3 protein putative” were found to be upregulated in the PRr strain. These proteins are capable of binding to numerous phosphorylated proteins including transcription factors, biosynthetic enzymes, cytoskeletal proteins, signaling molecules and display important anti-apoptotic characteristics [30].

### Chaperones and Stress Proteins

A number of chaperones and stress proteins showed increased expression levels in the PRr strain. Analysis of the data revealed upregulation in the levels in the PRr strain; namely three HSPs (>2 fold) and two chaperonins (>2 fold). All the three HSPs belong to the HSP70 family. Heat shock proteins have been shown to confer resistance against drugs [31].

### Proteins involved in Translation Machinery

Paromomycin is known to bind to the small subunit of ribosomes and inhibit protein synthesis [1]. Our data analysis revealed that three out of four significantly upregulated proteins are components of small ribosomal subunit. A total of 56 proteins involved in translation including ribosomal subunit proteins and translation factors were identified by SILAC. Upregulation of ribosomal proteins rpS26 (∼3.5 fold) and rpS6 (∼1.57 fold) was detected in the paromomycin resistant strain. These two proteins have also been found to be involved in self-translation regulation in humans. Ribosomal protein, rpS26 has been shown to be important for the normal growth of *S. cerevisiae* [32]. Ribosomal protein, rpL18a putative has been implicated in cell growth regulation and was seen to be upregulated (∼1.5 fold). Translational factor, EF3 was found to be downregulated in the paromomycin resistant strain (∼5.88 fold).

### Proteins Involved in DNA/RNA Processing

A few proteins involved in transcription were also identified including some of the histones which are important for DNA replication and nucleosome assembly. Basic transcription factor 3a (BTF3a) putative was found to be ∼3 fold upregulated. It has been observed that down-regulation of BTF3a is involved in inhibition of transcription and proteins synthesis in the apoptotic K562 cells [33]. It also acts as a transcriptional regulator and is associated with apoptosis and/or cell cycle arrest [34], [35]. Two of the nucleic acid associated proteins involved in RNA processing were found to be significantly down regulated in case of the PR resistant strain. Fibrillarin, a key small nucleolar protein in eukaryotes which plays an important role in ribosomal biogenesis was found to be ∼2.2 fold downregulated in the PR resistant strain [36]. Fibrillarin (NOP1) is highly conserved throughout evolution from unicellular organisms such as yeast and trypanosomes to humans [37]. It is involved in rRNA processing and modification in *Trypanosoma brucei* [38]. A NOP1 knockout is lethal in *S. cerevisiae* and mutants of this gene exhibit defects in pre-rRNA processing, modification and ribosome assembly [39].

### Drug Affinity Pull Down-Assay of the Paromomycin Interacting Proteins

**Figure 5:**
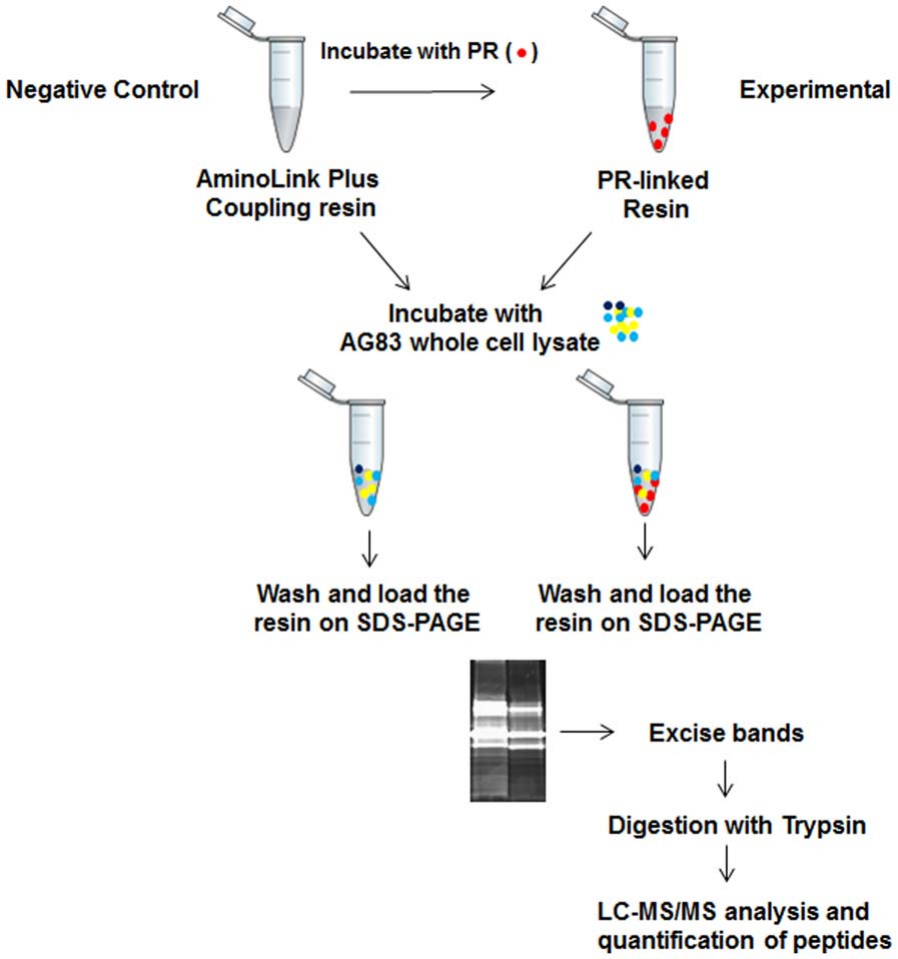
Overview of the affinity pull-down assay using PR-linked resin. Paromomycin was bound to AminoLink Plus coupling resin. The PR-linked resin (experimental) and unlinked resin (used as negative control) were incubated with AG83 whole cell lysate. The beads were then washed and the resin was loaded onto 10% SDS-PAGE gel. The gel was stained with sypro ruby and the fragments were excised and digested with trypsin. The protein samples were analyzed by LC-MS/MS and the results compared to determine the proteins that interacted with PR selectively.

The combination of drug affinity pull-down assays with in situ digestion and LC-MS/MS analysis is a useful tool in obtaining complex information about a primary drug target as well as its protein interactors. Pull down-assay using the PR linked resin followed by LC-MS/MS analysis revealed a number of proteins upon LC-MS/MS analysis which might be interacting with PR inside the cell. The basic scheme of the experiment is shown in Figure 5.The data obtained for PR linked and unlinked resins were compared to obtain the proteins that bound exclusively to the PR linked resin. The identified proteins that interacted with PR are listed in Table 3. Of particular interest were the paraflagellar rod proteins 1D and 2C, found only in the flagellum of the parasites [40]. Another protein, prohibitin was also found to interact with PR. Prohibitin in *Leishmania* is reported to be concentrated at the surface of the flagellar and the aflagellar pole [28]. The flagellar pocket membrane is the only part of the cell surface that supports endocytosis and exocytosis [41]. The present data further supports the role of such a process in drug uptake. Another protein, P-type H^+^ ATPase putative was also found to be interacting with PR. This protein is reported to be localized in the intracellular compartments and is involved in the acidification and forms a part of endocytic pathway in *Trypanosoma cruzi* [42]. It is possible that the PR interacts with this protein after it is endocytosed or sequestered in the vesicles by the parasite.

**Table 3:** Drug affinity pull-down assays with in situ digestion and LC-MS/MS analysis identified proteins from wild type *L. donovani* promastigotes that bind to paromomycin.

S. No.	Accession No.	Name of Protein	Number of Peptides	Probability	Percent coverage
1.	LinJ16_V31510, LinJ16_V31520	Paraflagellar rod protein 2C	2	1	4.3
2.	LinJ18_V3.1490, LinJ18_V3.1500, LinJ18_V3.1510	P-type H+-ATPase, putative	3	0.99	5.8
3.	LinJ29_V3.1880,LinJ29_V3.1890	Paraflagellar rod protein 1D	5	1	9.3
4.	LinJ31_V3.2680	RNA Polymerase II largest subunit	1	0.99	1.1
5.	LinJ35_V3.0070	Prohibitin	1	0.95	4.8

The protein coverage is based on the non-redundant peptides identified by PeptideProphet.

The results are representative of two independent experiments.

## Discussion

The control of leishmaniasis relies mainly on a few chemotherapeutic agents and emerging resistance towards these agents is one of the major clinical problems today. Paromomycin, an aminoglycoside antibiotic, is one of the drugs currently used in the chemotherapy of cutaneous and visceral leishmaniasis. Paromomycin inhibits protein synthesis by interacting with ribosomal RNA subunits [1]. However, its mode of action is not well understood in *Leishmania spp.* Reports suggest that paromomycin might act by interacting with the ribosomes or disrupting the mitochondrial membrane potential [14], [15]. In order to understand the mode of action of this antibiotic at the molecular level, we employed a quantitative proteomics technique using stable isotope labeling with amino acids in cell culture (SILAC) coupled with mass spectrometry to quantify changes in protein levels between paromomycin resistant (PRr) and sensitive cells.

We had earlier elucidated the effect and mechanism of uptake of paromomycin in *Leishmania donovani* [15]. Our earlier work suggests binding of the cationic paromomycin to the negatively charged leishmanial glycocalyx. In bacteria, aminoglycosides are reported to associate with lipopolysaccharide (LPS) and other anionic components of the bacterial cell surface [43]. Lipophosphoglycan (LPG), a highly negatively charged molecule is a major component of the cell surface of promastigotes of *Leishmania* [44], [45]. Several glycosylphosphatidylinositols have been reported to accumulate in the endosomes [46], [47]. We had earlier proposed that paromomycin associates with LPG at the leishmanial surface and also suggested other components may also be involved in binding. Our hypothesis was that after binding to the surface, internalization appears to proceed by endocytosis since a number of typical inhibitors of endocytosis reduced the rate of uptake of the drug [15]. Once inside the cell it is possible that the drug enters the mitochondrion. The uptake of this cationic agent in the mitochondria is dependent on the high membrane potential [15].

The present study was aimed to investigate the global proteome differences between the wild type AG83 strain and a paromomycin resistant (PRr) strain of *L. donovani*. Table 4 gives a brief summary of the classes of proteins that were altered in the PR resistant strain. The identified parasite proteins further support our earlier understanding of the mode of action and underlying mechanism of resistance to paromomycin in *Leishmania*. Proteome screens have been shown to be useful for identifying novel resistance mechanisms. The data revealed changes in the level of proteins involved in metabolic processes like glycolysis and those involved in other fundamental processes like translation, transcription, signal transduction, chaperones/stress, intracellular survival and pro-apoptosis.

**Table 4:** Modulation in expression of proteins in paromomycin -susceptible (AG83 WT) and -resistant (PRr) *Leishmania donovani.*

Cellular Function	Proteins modulated in the Paromomycin resistant strain
Intracellular Survival, Processing and Virulence	14-3-3 protein, LPG biosynthetic protein and IgE dependent histamine release factor were found to be upregulated. Prohibitin was found to be downregulated.
Vesicle trafficking	Proteins involved in vesicle trafficking, fusion and endocytosis like vesicle fusing ATPase and vacuolar ATP synthase were found to be upregulated.
Translation	Ribosomal proteins were found to be upregulated in the PR resistant strain. This may be because PR is known to inhibit translation in *E. coli*
Chaperones/stress proteins	Heat shock proteins and chaperonins were found to be upregulated. HSPs protect cell from toxic external stimuli and have been implicated in drug resistance.
Metabolism	Proteins involved in glycolysis were seen to be upregulated

Several of the proteins involved in the translational machinery, especially the ribosomal proteins, were found to be upregulated in the resistant strain (Table 1). In *Leishmania*, paromomycin has been implicated in targeting ribosomes directly and differential effects of paromomycin on the translation processes of the *Leishmania* parasite and its mammalian hosts has also been reported [48]. In light of this, our data on the up-regulation of the ribosomal proteins could be explained as one of the mechanisms utilized by the resistant parasites as a defence against paromomycin. Furthermore, our previous report also shows an overall less percent inhibition of protein synthesis by paromomycin in the PRr strain as compared to the WT [15].

Of the upregulated proteins in the PRr strain, LPG putative deserves special attention as it has been identified as a virulence factor in *Leishmania* [49]. Lipophosphoglycan biosynthetic protein, putative or *LPG3* is an ortholog of eukaryotic glucose regulated protein 94 (GRP 94). Its null mutants in *Leishmania* are viable but show severe defects in phosphoglycosylation. Its role has been found to be restricted to the synthesis of glycoconjugates implicated in parasite virulence, including LPG and other PG-bearing molecules and GPI-anchored proteins [49].

LPG biosynthetic protein, vesicle fusing ATPase and vacuolar ATPase were upregulated in the PR resistant strain. The ultra-structural studies of the paromomycin resistant strain revealed the presence of increased number of vesicular vacuoles in comparison to the sensitive wild type strain (Figure 4).

Affinity pull-down assay using PR linked resin identified proteins that interact with PR. Paraflagellar rod proteins and prohibitin were found to interact with paromomycin. Prohibitin in *Leishmania* is also reported to be concentrated at the surface of the flagellar and the aflagellar pole [28]. It is well known that the flagellar pocket facilitates internalization of the molecules. Hence binding of PR to the paraflagellar rod proteins and prohibitin is of significance in the present study.

**Figure 6:**
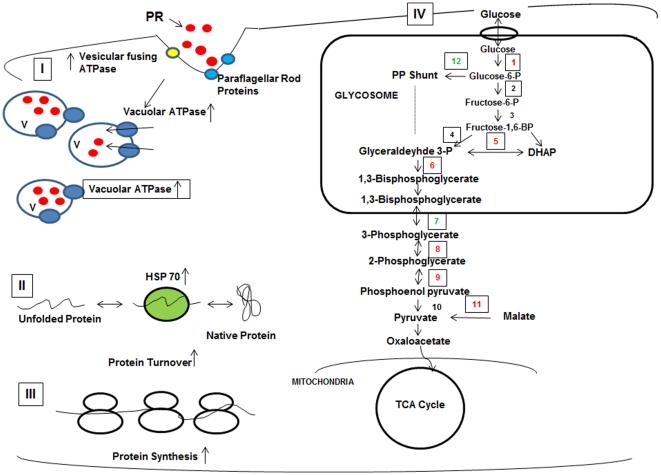
Proposed model for mechanism of paromomycin resistance in *L. donovani*. (I) The drug paromomycin (red circles) interacts with paraflagellar rod proteins (blue circles) and prefoldin (yellow circles) and is then taken up by the *Leishmania* promastigotes by endocytosis. PR is then sequestered in to the vacuoles. The pump, vacuolar ATPase is found to be upregulated and the number of vacuoles is increased in the resistant parasites. (II) The chaperone proteins are found to be upregulated in the resistant cells as a result of the stress caused by paromomycin and are also involved in increased protein turnover. (III) A number of ribosomal protein subunits were found to be regulated, increasing the total protein synthesis. (IV) The glycolytic pathway was found to be upregulated. Proteins marked in red represent the upregulated proteins and the ones marked in green represent the down- regulated proteins in the case of paromomycin resistant strain. Enzymes indicated: (**1**) Hexokinase, (**2**) Phosphoglucose isomerase, (**3**) Phosphofructokinase, (**4**) Aldolase (**5**) Triose phosphate isomerase, (**6**) Glyceraldehyde 3-phosphate dehydrogenase, (**7**) Phosphoglycerate kinase (**8**) 2,3-bisphosphoglycerate independent phosphoglycerate mutase, (**9**) Enolase, (**10**) Pyruvate kinase, (**11**) Malic enzyme (**12**) glucose 6-phosphate dehydrogenase.

In summary, it is clear that the molecular mechanisms contributing to paromomycin resistance comprise a complex multifactorial network. In view of our earlier and present studies we propose the following mode of action for PR in *L. donovani* (Figure 6). It is proposed that the drug binds to the paraflagellar rod proteins and prohibitin and then internalization appears to proceed by endocytosis since a number of typical inhibitors of endocytosis reduced the rate of uptake of the drug [15]. Another protein, P-type H^+^ ATPase putative was also found to interact with PR. This protein is localized in the intracellular compartments and is reported to be involved in acidification and forms a part of endocytic pathway in *Trypanosoma cruzi* [42]. It is possible that PR interacts with this protein after it is endocytosed or sequestered in the vesicles by the parasite. The vesicular sequestration of paromomycin into the internal vesicles by the parasite using fluorescent derivative of the aminoglycoside antibiotic, paromomycin would be required to show that the drug accumulates in the vesicles. It is possible that vesicular sequestration of paromomycin in to the vacuoles by these resistant parasites might be involved in conferring the resistance phenotype. Once inside the cell PR inhibits protein synthesis by targeting several of the proteins involved in the translational machinery, in particular the ribosomal proteins. Overall, this study characterized the mode of action and underlying mechanism of resistance to paromomycin in *Leishmania donovan*i. However, further investigation of vesicle-mediated mechanism of paromomycin resistance is presently going on in the laboratory to conclusively show drug sequestration into the internal vesicles of the resistant parasites.

## Materials and Methods

### Chemicals

The medium M199 deficient in L-lysine and L-arginine was custom synthesized from SAFC biosciences (MO, USA). The labeled amino acids, ^13^C_6_ L-lysine-HCl and ^13^C_6_^15^N_4_ L-arginine-HCl were obtained from Pierce Biotech (Illinois, USA). Dialyzed fetal bovine serum was purchased from Invitrogen (CA, USA). Mass spec (MS) grade trypsin was purchased from Promega (WI, USA). AminoLink coupling resin was purchased from Pierce Biotech (IL, USA). All the solvents used for preparation and running of samples were of MS grade and were available commercially.

### Parasite and Culture Conditions

Promastigotes of *Leishmania donovani* clones, AG83 (MHOM/IN/80/AG83) wild-type (WT) and a paromomycin resistant strain (PRr) were used in the present study. PRr strain used in this study was raised in the laboratory as reported [15]. PRr strain had an IC_50_ of 145 µM and was three-fold resistant to paromomycin compared to the wild-type [15]. Promastigotes were routinely cultured at 22°C in modified M-199 medium (Sigma, St. Louis. MO, USA) supplemented with 10% heat-inactivated fetal bovine serum (FBS; Gibco/BRL, Life Technologies Scotland, UK) and 0.13 mg/mL penicillin and streptomycin.

### SILAC Labeling of Cells

For SILAC analysis, promastigotes were grown in custom synthesized media, M199 depleted of L-lysine and L-arginine (Sigma, USA) supplemented with 10% dialyzed fetal bovine serum (Invitrogen, Carlsbad, CA, USA), antibiotics and one of the two SILAC media formulations at 22°C [normal isotopic abundance L-lysine (70 mg/L) and L-arginine (70 mg/L)] for the wild type and ^13^C_6_ L-lysine-HCl and ^13^C_6_^15^N_4_ L-arginine-HCl (Pierce Biotechnology) for the PRr parasites] (Fig. 1). A regular sub-culturing in this medium was done for at least six cell divisions in order to achieve 100% labeling of the cellular proteins before analysis.

### Preparation of Protein Samples

The cells were harvested, washed with 1X PBS and lysed. The protein was quantitated by Bradford's method [50] and equal amount of protein (100 µg) from both the cell lysates was mixed and separated by 10% SDS-PAGE and stained with Sypro Ruby stain (Bio-Rad). The entire gel lane was cut into 10 sections for in-gel digestion. Each section was further cut into small pieces of approximately 1 mm each. The gel pieces were dehydrated in the presence of 50% acetonitrile and then rehydrated with 50 mM ammonium bicarbonate. Digestion of the samples was carried out using sequencing grade modified trypsin (Promega) (12.5 ng/µl) in 50 mM ammonium bicarbonate at 37°C overnight. The tubes were then centrifuged and supernatant collected. The peptides were extracted from the gel using 20 mM ammonium bicarbonate, followed by extraction with 5% formic acid in 50% acetonitrile. The samples were dried in speedvac and stored at −80°C till further analysis.

### Liquid Chromatography–Mass Spectrometry (LC-MS/MS), Data Base Analysis and Relative Quantification

LC-MS/MS was performed using LTQ linear ion trap mass spectrometer (Thermo Fisher Scientific). The LC system consisted of a fused-silica nanospray needle packed in-house with C18 reverse-phase material. The peptide samples were loaded onto the reversed phase column using a two-mobile-phase solvent system consisting of 0.4% acetic acid in water (A) and 0.4% acetic acid in acetonitrile (B) at a flow rate of 200 µl/min. The mass spectrometer operated in a data-dependent MS/MS mode over the *m/z* range of 400–2000. For each cycle, the five most abundant ions from each MS scan were selected for MS/MS analysis using 45% normalized collision energy. Dynamic exclusion was used to exclude ions that had been detected twice in a 30 sec window for 3 min. Raw MS/MS data were submitted to Bioworks 3.3 (ThermoElectron, San Jose, CA, USA) and searched using the SEQUEST algorithm against *L. infantum* protein database ver 3.0 (ftp://ftp.sanger.ac.uk/pub/pathogens/L_infantum/). The SEQUEST output files were analyzed and validated by PeptideProphet. Proteins and peptides with a probability score of ≥0.9 were accepted. The quantification was based on two independent SILAC and LC-MS/MS experiments.

### Transmission Electron Microscopy

Cells were fixed in a solution of 2% paraformaldehyde and 2.5 mM CaCl_2_ in 0.1 M sodium cacodylate buffer pH 7.2, post-fixed in 1% osmium tetroxide and 0.8% potassium ferricyanide in the same buffer, dehydrated in an acetone series and embedded in Polybed resin. Thin sections were stained with uranyl acetate and lead citrate and observed under a FEI Morgagni™ transmission electron microscope. Images are representative of 2 independent experiments.

### Affinity Pull-Down of the Paromomycin Interacting Proteins

Paromomycin (PR) was linked to AminoLink plus coupling resin (Pierce Biotechnology) according to the manufacturer's protocol (the resin contains aldehyde groups that react with primary amines present in paromomycin to form Schiff's base bonds). The unbound paromomycin was removed by centrifuging the resin at 2000 rpm and then washing with 1 X phosphate buffered saline (PBS), pH 7.2. The binding efficiency of the beads with PR was found to be 66%. Wild-type AG83 cells (1×10^8^) were harvested and lysed with lysis buffer (10 mM Tris.Cl pH 7.2, 10 mM MgCl_2_, 150 mM KCl, 0.1% Triton-X 100, 1% BSA and protease inhibitor cocktail). The lysate was incubated with paromomycin-linked agarose beads for 2 h at 4°C with continuous mixing. The suspension was then centrifuged at 2000 rpm for 10 min at 4°C and the beads were transferred to a fresh tube. The beads were washed four times with a wash buffer (10 mM Tris.Cl pH 7.2, 10 mM MgCl_2_, 150 mM KCl and 0.1% Triton-X 100) and finally resuspended in 1X SDS PAGE dye and loaded onto 10% SDS-PAGE gel. The gel was stained with Sypro Ruby stain and the bands were visualized under UV and desired bands were cut and subjected to the LC-MS/MS analysis in a 4000 Q TRAP LC-MS/MS system (Applied Bisosytems MDS SCIEX) for peptide ionization and detection. The SEQUEST output files were analyzed and validated by PeptideProphet. Proteins and peptides with a probability score of ≥0.9 were accepted. Beads not linked with paromomycin were used as negative control. The results are representative of two independent experiments.

## Supporting Information

Table S1 **List of proteins identified in Set A and Set B at ≥95% confidence.**
(XLS)

Table S2 **Proteins identified using SILAC and categorized on the basis of function quantitated at >95% confidence and having ≥2 peptides.**
(XLS)

## References

[1] Davis BD (1987). Mechanism of bactericidal action of aminoglycosides.. Microbiol Rev.

[2] Gillin FD, Diamond LS (1981). Inhibition of clonal growth of Giardia lamblia and Entamoeba histolytica by metronidazole, quinacrine, and other antimicrobial agents.. J Antimicrob Chemother.

[3] Flanigan TP, Ramratnam B, Graeber C, Hellinger J, Smith D (1996). Prospective trial of paromomycin for cryptosporidiosis in AIDS.. Am J Med.

[4] Hirokawa G, Kaji H, Kaji A (2007). Inhibition of antiassociation activity of translation initiation factor 3 by paromomycin.. Antimicrob Agents Chemother.

[5] Fourmy D, Recht MI, Blanchard SC, Puglisi JD (1996). Structure of the A site of Escherichia coli 16S ribosomal RNA complexed with an aminoglycoside antibiotic.. Science.

[6] Khalil EA, el Hassan AM, Zijlstra EE, Hashim FA, Ibrahim ME (1998). Treatment of visceral leishmaniasis with sodium stibogluconate in Sudan: management of those who do not respond.. Ann Trop Med Parasitol.

[7] Sundar S, More DK, Singh MK, Singh VP, Sharma S (2000). Failure of pentavalent antimony in visceral leishmaniasis in India: report from the center of the Indian epidemic.. Clin Infect Dis.

[8] Lira R, Sundar S, Makharia A, Kenney R, Gam A (1999). Evidence that the high incidence of treatment failures in Indian kala-azar is due to the emergence of antimony-resistant strains of Leishmania donovani.. J Infect Dis.

[9] Armijos RX, Weigel MM, Calvopina M, Mancheno M, Rodriguez R (2004). Comparison of the effectiveness of two topical paromomycin treatments versus meglumine antimoniate for New World cutaneous leishmaniasis.. Acta Trop.

[10] Thakur CP, Kanyok TP, Pandey AK, Sinha GP, Messick C (2000). Treatment of visceral leishmaniasis with injectable paromomycin (aminosidine). An open-label randomized phase-II clinical study.. Trans R Soc Trop Med Hyg.

[11] Sundar S, Jha TK, Thakur CP, Sinha PK, Bhattacharya SK (2007). Injectable paromomycin for Visceral leishmaniasis in India.. N Engl J Med.

[12] el-On J, Halevy S, Grunwald MH, Weinrauch L (1992). Topical treatment of Old World cutaneous leishmaniasis caused by Leishmania major: a double-blind control study.. J Am Acad Dermatol.

[13] Scott JA, Davidson RN, Moody AH, Grant HR, Felmingham D et al (1992). Aminosidine (paromomycin) in the treatment of leishmaniasis imported into the United Kingdom.. Trans R Soc Trop Med Hyg.

[14] Maarouf M, de KY, Brown S, Petit PX, Robert-Gero M (1997). In vivo interference of paromomycin with mitochondrial activity of Leishmania.. Exp Cell Res.

[15] Jhingran A, Chawla B, Saxena S, Barrett MP, Madhubala R (2009). Paromomycin: uptake and resistance in Leishmania donovani.. Mol Biochem Parasitol.

[16] Fernandez MM, Malchiodi EL, Algranati ID (2011). Differential effects of paromomycin on ribosomes of Leishmania mexicana and mammalian cells.. Antimicrob Agents Chemother.

[17] Rosenzweig D, Smith D, Opperdoes F, Stern S, Olafson RW (2008). Retooling Leishmania metabolism: from sand fly gut to human macrophage.. FASEB J.

[18] Drummelsmith J, Brochu V, Girard I, Messier N, Ouellette M (2003). Proteome mapping of the protozoan parasite Leishmania and application to the study of drug targets and resistance mechanisms.. Mol Cell Proteomics.

[19] Walker J, Acestor N, Gongora R, Quadroni M, Segura I (2006). Comparative protein profiling identifies elongation factor-1beta and tryparedoxin peroxidase as factors associated with metastasis in Leishmania guyanensis.. Mol Biochem Parasitol.

[20] Dea-Ayuela MA, Rama-Iniguez S, Bolas-Fernandez F (2006). Proteomic analysis of antigens from Leishmania infantum promastigotes.. Proteomics.

[21] Vergnes B, Gourbal B, Girard I, Sundar S, Drummelsmith J (2007). A proteomics screen implicates HSP83 and a small kinetoplastid calpain-related protein in drug resistance in Leishmania donovani clinical field isolates by modulating drug-induced programmed cell death.. Mol Cell Proteomics.

[22] van Weelden SW, van Hellemond JJ, Opperdoes FR, Tielens AG (2005). New functions for parts of the Krebs cycle in procyclic Trypanosoma brucei, a cycle not operating as a cycle.. J Biol Chem.

[23] Vila-Sanjurjo A, Squires CL, Dahlberg AE (1999). Isolation of kasugamycin resistant mutants in the 16 S ribosomal RNA of Escherichia coli.. J Mol Biol.

[24] Kramer DL, Black JD, Mett H, Bergeron RJ, Porter CW (1998). Lysosomal sequestration of polyamine analogues in Chinese hamster ovary cells resistant to the S-adenosylmethionine decarboxylase inhibitor, CGP-48664.. Cancer Res.

[25] Shapiro AB, Fox K, Lee P, Yang YD, Ling V (1998). Functional intracellular P-glycoprotein.. Int J Cancer.

[26] Sognier MA, Zhang Y, Eberle RL, Sweet KM, Altenberg GA (1994). Sequestration of doxorubicin in vesicles in a multidrug-resistant cell line (LZ-100).. Biochem Pharmacol.

[27] Legare D, Richard D, Mukhopadhyay R, Stierhof YD, Rosen BP (2001). The Leishmania ATP-binding cassette protein PGPA is an intracellular metal-thiol transporter ATPase.. J Biol Chem.

[28] Jain R, Ghoshal A, Mandal C, Shaha C (2010). Leishmania cell surface prohibitin: role in host-parasite interaction.. Cell Microbiol.

[29] Welburn SC, Murphy NB (1998). Prohibitin and RACK homologues are up-regulated in trypanosomes induced to undergo apoptosis and in naturally occurring terminally differentiated forms.. Cell Death Differ.

[30] Dougherty MK, Morrison DK (2004). Unlocking the code of 14-3-3.. J Cell Sci.

[31] Brochu C, Haimeur A, Ouellette M (2004). The heat shock protein HSP70 and heat shock cognate protein HSC70 contribute to antimony tolerance in the protozoan parasite leishmania.. Cell Stress Chaperones.

[32] Wu M, Tan HM (1994). The Saccharomyces cerevisiae homologue of ribosomal protein S26.. Gene.

[33] Li R, Liu XL, Du QF, Zhang S, Luo RC (2004). [Proteome analysis of apoptotic K562 cells induced by harringtonine].. Zhonghua Xue Ye Xue Za Zhi.

[34] Kusumawidjaja G, Kayed H, Giese N, Bauer A, Erkan M (2007). Basic transcription factor 3 (BTF3) regulates transcription of tumor-associated genes in pancreatic cancer cells.. Cancer Biol Ther.

[35] Brockstedt E, Otto A, Rickers A, Bommert K, Wittmann-Liebold B (1999). Preparative high-resolution two-dimensional electrophoresis enables the identification of RNA polymerase B transcription factor 3 as an apoptosis-associated protein in the human BL60-2 Burkitt lymphoma cell line.. J Protein Chem.

[36] Amin MA, Matsunaga S, Ma N, Takata H, Yokoyama M (2007). Fibrillarin, a nucleolar protein, is required for normal nuclear morphology and cellular growth in HeLa cells.. Biochem Biophys Res Commun.

[37] Jansen RP, Hurt EC, Kern H, Lehtonen H, Carmo-Fonseca M (1991). Evolutionary conservation of the human nucleolar protein fibrillarin and its functional expression in yeast.. J Cell Biol.

[38] Barth S, Shalem B, Hury A, Tkacz ID, Liang XH (2008). Elucidating the role of C/D snoRNA in rRNA processing and modification in Trypanosoma brucei.. Eukaryot Cell.

[39] Tollervey D, Lehtonen H, Jansen R, Kern H, Hurt EC (1993). Temperature-sensitive mutations demonstrate roles for yeast fibrillarin in pre-rRNA processing, pre-rRNA methylation, and ribosome assembly.. Cell.

[40] de SW, Souto-Padron T (1980). The paraxial structure of the flagellum of trypanosomatidae.. J Parasitol.

[41] Overath P, Stierhof YD, Wiese M (1997). Endocytosis and secretion in trypanosomatid parasites - Tumultuous traffic in a pocket.. Trends Cell Biol.

[42] Vieira M, Rohloff P, Luo S, Cunha-e-Silva NL, de SW (2005). Role for a P-type H+-ATPase in the acidification of the endocytic pathway of Trypanosoma cruzi.. Biochem J.

[43] Magnet S, Blanchard JS (2005). Molecular insights into aminoglycoside action and resistance.. Chem Rev.

[44] Guha-Niyogi A, Sullivan DR, Turco SJ (2001). Glycoconjugate structures of parasitic protozoa.. Glycobiology.

[45] Ilgoutz SC, McConville MJ (2001). Function and assembly of the Leishmania surface coat.. Int J Parasitol.

[46] Zheng Z, Tweten RK, Mensa-Wilmot K (2005). Intracellular glycosylphosphatidylinositols accumulate on endosomes: toxicity of alpha-toxin to Leishmania major.. Eukaryot Cell.

[47] Glaser TA, Baatz JE, Kreishman GP, Mukkada AJ (1988). pH homeostasis in Leishmania donovani amastigotes and promastigotes.. Proc Natl Acad Sci U S A.

[48] Maarouf M, Lawrence F, Croft SL, Robert-Gero M (1995). Ribosomes of Leishmania are a target for the aminoglycosides.. Parasitol Res.

[49] Descoteaux A, Avila HA, Zhang K, Turco SJ, Beverley SM (2002). Leishmania LPG3 encodes a GRP94 homolog required for phosphoglycan synthesis implicated in parasite virulence but not viability.. EMBO J.

[50] Bradford MM (1976). A rapid and sensitive method for the quantitation of microgram quantities of protein utilizing the principle of protein-dye binding.. Anal Biochem.

